# Irreversible Electroporation Treatment With Intraoperative Biliary Stenting for Unresectable Perihilar Cholangiocarcinoma: A Pilot Study

**DOI:** 10.3389/fonc.2021.710536

**Published:** 2021-06-30

**Authors:** Po-Chih Yang, Yan-Jun Chen, Xiao-Yong Li, Chih-Yang Hsiao, Bing-Bing Cheng, Yu Gao, Bai-Zhong Zhou, Sheng-Yang Chen, Shui-Quan Hu, Quan Zeng, Kai-Wen Huang

**Affiliations:** ^1^ Graduate Institute of Clinical Medicine, College of Medicine, National Taiwan University, Taipei, Taiwan; ^2^ Department of Surgery, Fu Jen Catholic University Hospital, Fu Jen Catholic University, New Taipei City, Taiwan; ^3^ School of Medicine, Fu Jen Catholic University, New Taipei City, Taiwan; ^4^ Department of Hepatopancreatobiliary Surgery, The Fifth Affiliated Hospital, Zhengzhou University, Zhengzhou, China; ^5^ Department of Traumatology, National Taiwan University Hospital, Taipei, Taiwan; ^6^ Department of Surgery & Hepatitis Research Center, National Taiwan University Hospital, Taipei, Taiwan; ^7^ Endoscopic Center, the Fifth Affiliated Hospital, Zhengzhou University, Zhengzhou, China

**Keywords:** irreversible electroporation, perihilar cholangiocarcinoma (PHCCA), biliary stent, unresectable abdominal neoplasms, Jaundice cholangitis

## Abstract

**Background:**

Treating perihilar cholangiocarcinoma (PHCC) is particularly difficult due to the fact that it is usually in an advanced stage at the time of diagnosis. Irreversible electroporation treatment (IRE) involves the local administration of a high-voltage electric current to target lesions without causing damage to surrounding structures. This study investigated the safety and efficacy of using IRE in conjunction with intraoperative biliary stent placement in cases of unresectable PHCC.

**Methods:**

This study enrolled 17 patients with unresectable Bismuth type III/IV PHCC who underwent IRE in conjunction with intraoperative biliary stent placement (laparotomic) in two medical centers in Asia between June 2015 and July 2018. Analysis focused on the perioperative clinical course, the efficacy of biliary decompression, and outcomes (survival).

**Results:**

Mean total serum bilirubin levels (mg/dL) on postoperative day (POD) 7, POD30, and POD90 were significantly lower than before IRE (respectively 3.46 *vs* 4.54, p=0.007; 1.21 vs 4.54, p<0.001; 1.99 *vs* 4.54, p<0.001). Mean serum carbohydrate antigen 19-9 (CA19-9, U/ml) levels were significantly higher on POD3 than before the operation (518.8 *vs* 372.4, p=0.001) and significantly lower on POD30 and POD90 (respectively 113.7 *vs* 372.4, p<0.001; 63.9 *vs* 372.4, p<0.001). No cases of Clavien-Dindo grade III/IV adverse events or mortality occurred within 90 days post-op. The median progression-free survival was 21.5 months, and the median overall survival was 27.9 months. All individuals who survived for at least one year did so without the need to carry percutaneous biliary drainage (PTBD) tubes.

**Conclusions:**

It appears that IRE treatment in conjunction with intraoperative biliary stent placement is a safe and effective approach to treating unresectable PHCC. The decompression of biliary obstruction without the need for PTBD tubes is also expected to improve the quality of life of patients.

## Introduction

Cholangiocarcinoma is the second most common liver-related malignancy, second only to hepatocellular carcinoma. Due to its silent clinical characteristics, the disease has usually progressed to an advanced stage at the time of diagnosis, by which point it presents as obstructive jaundice (1). Cholangiocarcinoma is categorized according to the anatomical location of tumors as perihilar cholangiocarcinoma (PHCC), distal cholangiocarcinoma (DCC), or intrahepatic cholangiocarcinoma (ICC). PHCC is the most common, accounting for approximately 50% of all cases (2). At present, complete surgical resection remains the treatment option with the highest probability of long-term survival (3). Note however that the most common macroscopic growth pattern of PHCC involves periductal infiltration longitudinally along the biliary tract, commonly extending to the second- or third-order bile ducts (4). Note also that this growth pattern makes the R0 resection of PHCC exceedingly difficult or in many cases impossible. Radial invasion into the hepatic artery and portal vein adjacent to the biliary tract, even after aggressive treatment, can significantly reduce the duration of survival (5). In the event that R0 resection cannot be achieved, PHCC is deemed unresectable, whereupon palliative biliary decompression is used to relieve the symptoms of jaundice *via* drainage (external or internal) and prolong survival. It is important to note that the quality of life experienced by patients with unresectable PHCC is greatly hindered by the need to carry and constantly replace drainage tubes.

Irreversible electroporation (IRE) is a tumor ablation procedure in which a high-voltage electric current is used to alter the permeability of the cell membrane and thereby induce apoptosis. The fact that this procedure does not generate heat allows it to preserve the integrity of connective tissue in the ablation zone without compromising the efficacy of treatment. IRE has proven to be safe and effective for the treatment of locally advanced pancreatic cancer near the distal extrahepatic bile duct (6–8). However, there has been relatively little research on the use of IRE in the proximal extrahepatic bile duct for unresectable PHCC. Martin et al. first reported on the use of IRE for unresectable PHCC in conjunction with percutaneous biliary drainage (PTBD) (9). Note that even after the procedure, most of the patients in that study still had to carry PTBD tubes.

The current study examined the use of IRE in conjunction with intraoperative biliary stent placement to treat unresectable Bismuth type III/IV PHCC (10) in two medical centers in Asia. Analysis focused on the perioperative course of the procedure, the need for PTBD, and survival outcomes.

## Materials and Methods

This prospective study analyzed clinical data collected under the Eastern Ablation Registry Programme for Solid Tumor (EAST), focusing on patients with unresectable PHCC who had received IRE in conjunction with intraoperative biliary stent placement at National Taiwan University Hospital and the Fifth Affiliated Hospital of Zhengzhou University between June 2015 and July 2018. To identify the location and extension of the tumors, all patients underwent preoperative radiological examinations, including contrast-enhanced computed tomography (CT), magnetic resonance imaging (MRI), and cholangiography. Serological tumor markers and pathological evidence of cholangiocarcinoma from biopsy through endoscopic retrograde cholangiography (ERC) were used to confirm the diagnosis of Bismuth type III/IV PHCC. The criteria by which tumors were deemed unresectable were as follows: 1) Liver remnants after radical resection due to extensive proximal tumor invasion would be insufficient for survival; 2) Radial tumor invasion into the hepatic artery and/or portal vein precluded vascular reconstruction; and 3) R0 resection was impossible. Patients presenting distant metastasis, those with cardiac pacemakers, and those deemed intolerable to general anesthesia were excluded. The study was approved by the local institutional review boards of both medical centers, and all patients provided written informed consent (IRB number: 201210007DIC, ClinicalTrials.gov Identifier: NCT03362749).

All patients with preoperative obstructive jaundice in this study underwent biliary decompression *via* PTBD or endoscopic retrograde biliary drainage (ERBD). Patients without evidence of biliary infection or cholangitis proceeded directly to IRE.

**Figure 1:**
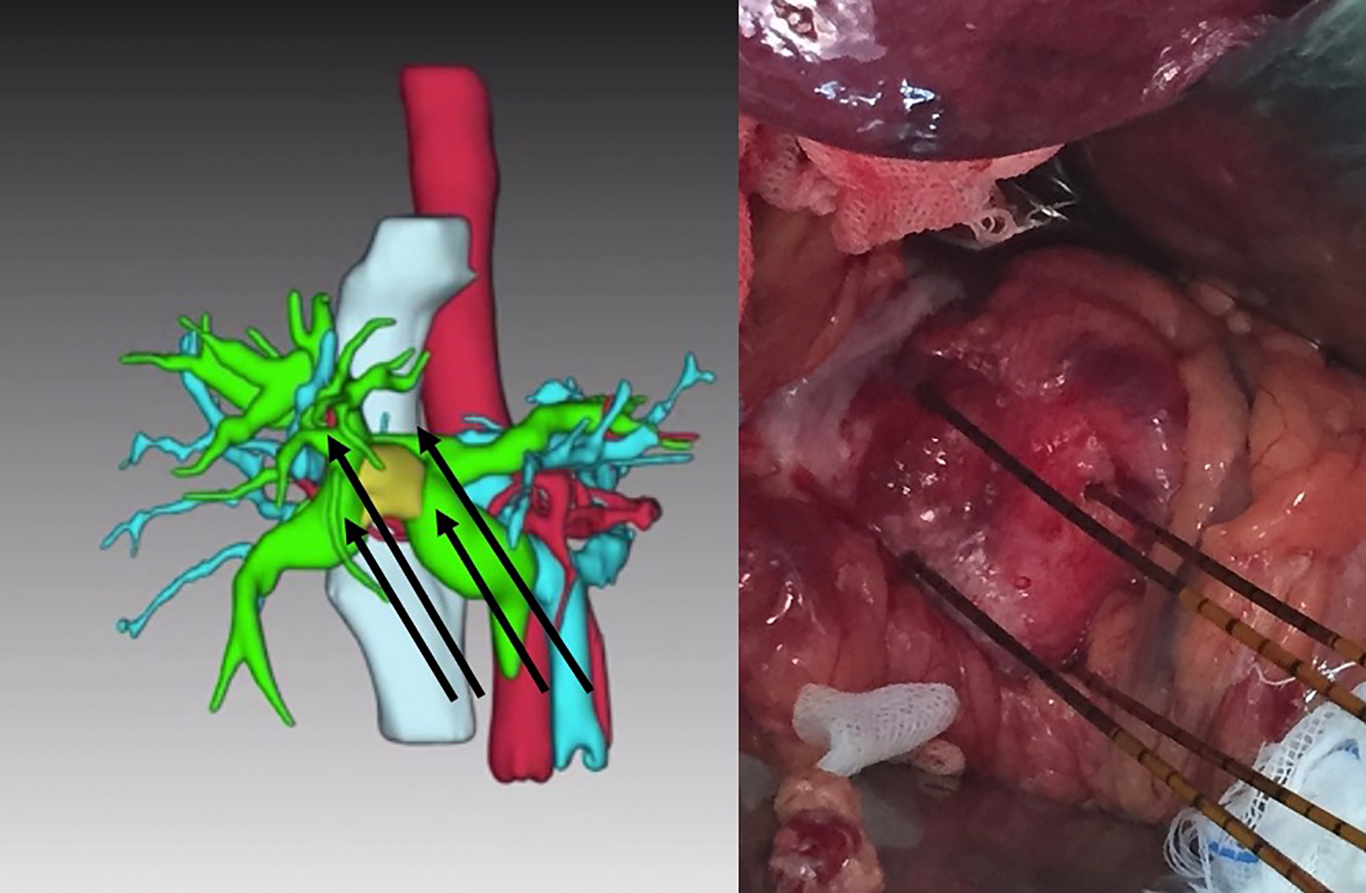
Four electrodes placed parallel to the hepatoduodenal ligament to embrace the tumor (yellow target in left image) in a caudal to cranial direction for the treatment of unresectable Bismuth type IIIA perihilar cholangiocarcinoma.

IRE was performed under general anesthesia and complete muscular blockade in accordance with established protocols (7, 8). All procedures in this study were implemented using the Nanoknife IRE system (AngioDynamics, Queensbury, USA) using the laparotomic approach. Following cholecystectomy, intraoperative ultrasonography was used to identify the location of the tumor and vascular anatomy in the hepatoduodenal ligament. Three or four electrodes were applied in a caudal-cranial direction (i.e., nearly parallel to the hepatoduodenal ligament) to prevent major vascular injury. The distance between electrodes ranged from 1.5 cm to 2.5 cm in accordance with the volume of the ablation zone with the aim of fully covering the tumor and enlarged hilar lymph nodes (**Figure 1**). Following the completion of IRE, the common bile duct was opened at the level of the cystic duct to allow the introduction of one 7-Fr. or 10-Fr. biliary drainage stent (Medi-Globe GmbH, Achenmuhle, Germany) into the biliary tract. The proximal end of this stent extended beyond the location of the tumor, and the distal end extended through the sphincter of the Oddi into the duodenum. Dissection of the hilar lymph nodes was also performed.

**Figure 2:**
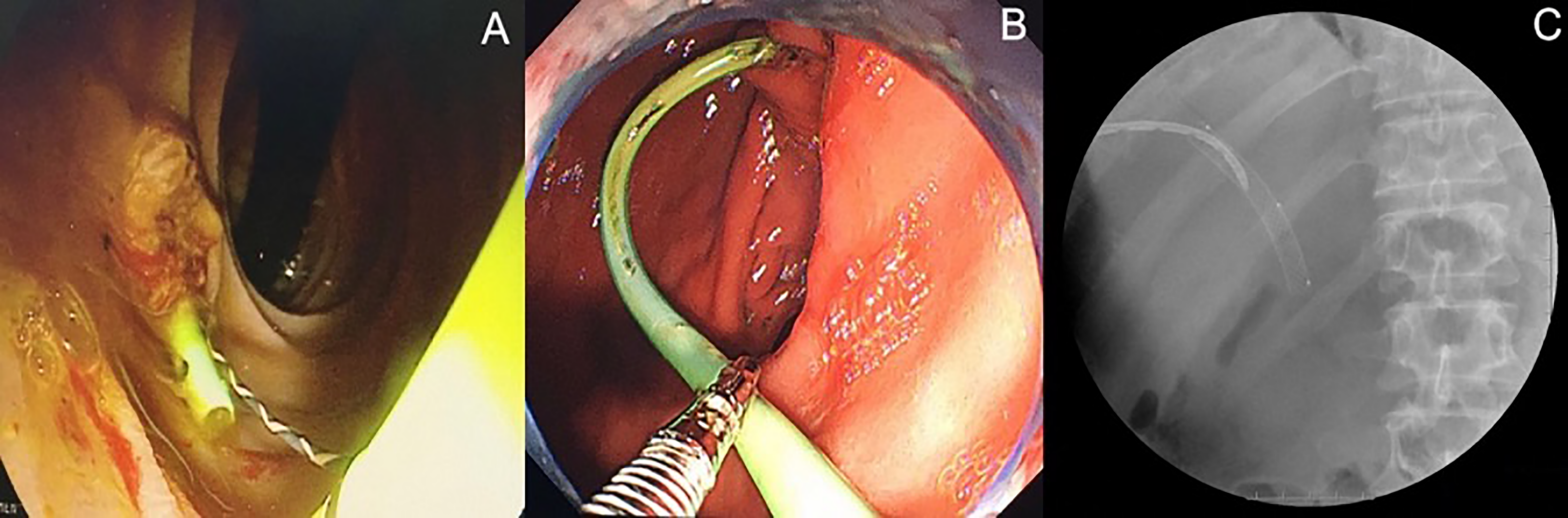
Endoscopic retrograde biliary drainage (ERBD) stents were replaced by expandable fully-covered metallic stents *via* endoscopic retrograde cholangiography (ERC) at three months after operation. **(A)** ERBD stent in *situ*. **(B)** ERBD stent removed by endoscopic snare. **(C)** ERC after insertion of metallic stents into extrahepatic bile duct.

Serological examinations, including bilirubin and carbohydrate antigen 19-9 (CA19-9), were routinely performed on postoperative day (POD) 1, 3, 7, 30, and 90. Cholangiography *via* the PTBD tube or intrabiliary stent was performed on POD7 to evaluate the patency of the biliary tract. As long as there was no evidence of biliary obstruction, PTBD tubes were removed on POD7. Follow-up radiological examinations, including contrast-enhanced CT scan and MRI, were conducted at 1 month and 3 months after IRE. Complete ablation was defined as no evidence of lesions in the previous tumor location in contrast-enhanced images. At 3 months after the operation, intrabiliary stents placed during the operation were replaced by expandable fully-covered metallic stents (WallFlex™, Boston Scientific Corp., USA) *via* ERC (**Figure 2**). Regular radiological and serological examinations were performed at intervals of 3 months. Local tumor progression was defined as the development of new lesions in the biliary tract with elevated serum CA19-9 levels. Occluded biliary metallic stents were replaced *via* ERC to relieve biliary obstruction. All patients have received adjuvant systemic therapy according to standard recommendation including Gemcitabine/Cisplatin (Oxaliplatin) and TS-1(Tegafur、Gimeracil、Oteracil potassium).

Continuous variables were expressed as the median and compared using paired samples t-test. Survival analysis was performed using the Kaplan-Meier method. A two-tailed p value of less than 0.05 was considered statistically significant. All analyses were conducted using STATA version 15 (StataCorp LLC, Texas, USA).

## Results

### Perioperative Outcomes

The median follow-up period for the 17 patients enrolled in this study was 24.5 months. The distribution of Bismuth classification was as follows: type IIIA (3), IIIB (1), and IV (13). According to the 8^th^ edition of the American Joint Committee on Cancer staging system (11), 10 patients had stage IIIB PHCC, while 7 patients had stage IIIC. There was no distant metastasis or ascites in all patients. Of the 17 patients, 15 patients underwent preoperative unilateral or bilateral PTBD (unilateral/bilateral: 12/3) due to obstructive jaundice (**Table 1**). The median duration of the IRE procedure was 300 minutes (range: 50-610 minutes). Intraoperative hilar lymph node dissection revealed regional metastasis in 16 patients. Two cases of Clavien-Dindo grade (C-D grade) II atrial fibrillation, one case of C-D grade II upper gastrointestinal tract hemorrhage, and one case of C-D grade II biliary fistula developed after IRE. No C-D grade III or IV adverse events occurred. After cholangiography confirmation of biliary patency, PTBD tubes were removed from 16 patients on POD7; however, one patient presented with a postoperative biliary fistula. None of the patients died within 90 days after IRE.

**Table 1:** Baseline characteristics of patients before IRE.

Variable	IRE (n=17)
Age (y), median (range)	65 (41-83)
Sex (male/female), n	8/9
BMI (kg/m^2^), mean ± SD	23.6 ± 3.6
Albumin (g/dL), median (range)	3.3 (2.3-4.1)
Total bilirubin (mg/dL), median (range)	4.9 (0.8-9)
CA19-9 (U/mL), median (range)	336.8 (10-1153)
AST (U/L), median (range)	91 (17-622)
ALT (U/L), median (range)	101 (6-563)
PTBD, n (%)	15 (88)
Bismuth Type, n (%)	
Type IIIA/IIIB	3/1 (18/6)
Type IV	13 (76)

BMI, body mass index; CA19-9, carbohydrate antigen 19-9; AST, aspartate transferase; ALT, alanine transferase; PTBD, percutaneous biliary drainage.

### Changes in the Level of Bilirubin and CA19-9

**Figure 3:**
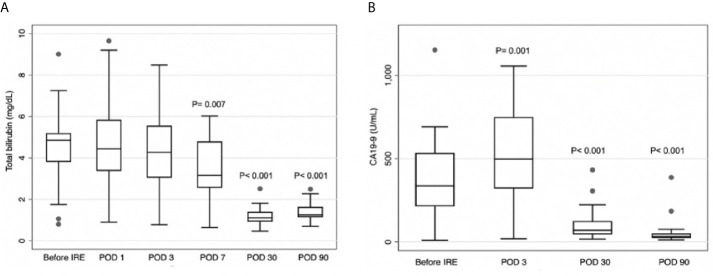
**(A)** Total serum total bilirubin levels on postoperative day (POD) 7, POD30, and POD90 were significantly lower than prior to irreversible electroporation treatment (IRE). **(B)** Serum carbohydrate antigen CA19-9 levels were higher on POD3 and lower on POD30 and POD90, compared to before IRE.

The mean serological total bilirubin level was significantly lower on POD7, POD30, and POD90, compared with preoperative levels (respectively 3.46 *vs*. 4.54, p=0.007; 1.21 *vs*. 4.54, p<0.001; 1.99 *vs*. 4.54, p<0.001) (**Figure 3A**). After IRE, mean serological CA19-9 levels were significantly higher on POD3 (518.8 *vs*. 372.4; p=0.001) but significantly lower on POD30 and POD90, compared with preoperative levels (respectively 113.7 *vs*. 372.4, p<0.001; 63.9 *vs*. 372.4, p<0.001) (**Figure 3B**).

### Short-Term Oncological Outcomes

**Figure 4:**
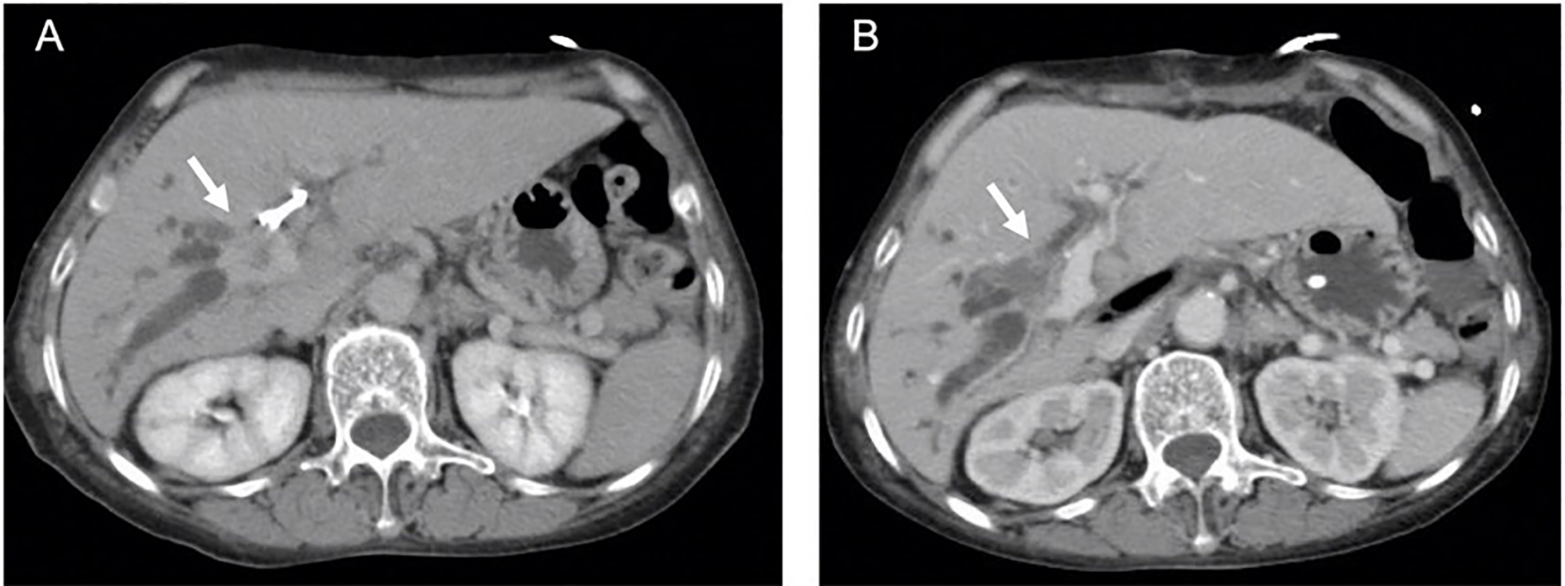
**(A)** Arrow indicates enhanced Bismuth type IIIA perihilar cholangiocarcinoma (PHCC) with percutaneous biliary drainage (PTBD) tube in computed tomography (CT) scan. **(B)** No evidence of enhanced tumor (arrow) with patent biliary tract was observed in CT scan at one month after irreversible electroporation treatment (IRE).

**Figure 5:**
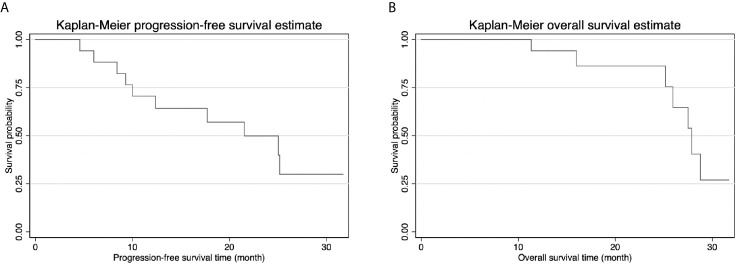
**(A)** Kaplan-Meier progression-free survival (PFS) curve showed median PFS was 21.5 months. **(B)** Kaplan-Meier overall survival curve (OS) curve showed median OS was 27.9 months.

Follow-up CT scans obtained on POD30 revealed no evidence of tumor enhancement in 16 of the patients (**Figure 4**), resulting in a complete ablation rate of 94.1%. Within one year after IRE, three patients (17.6%) developed local recurrence in the hepatic hilum, and four patients (23.5%) presented distant metastasis. The 1-year overall survival rate was 94.1%, and none of the survivors required PTBD tubes during that period (**Table 2**). The median progression-free survival (PFS) duration was 21.5 months (95% CI=10.8-32.2), and the median overall survival (OS) duration was 27.9 months (95% CI=25.3-30.5) (**Figure 5**). Most of the deaths during the follow-up period were due to distant metastasis, including intrahepatic metastasis, peritoneal metastasis, and multiple metastasis. Only three patients presented local recurrence.

**Table 2:** Outcome of patients at one year after IRE.

Variable	IRE (n=17)
Survival, n (%)	16 (94.1)
Regional lymphadenopathy, n (%)	3 (17.6)
Distant metastasis, n (%)	4 (23.5)
Internal stent, n (%)	15 (88.2)
PTBD, n	0
Drainage-free, n (%)	2 (11.8)

All variables are presented as number (%).

IRE, irreversible electroporation treatment; PTBD, percutaneous biliary drainage.

## Discussion

Patients with unresectable PHCC must commonly endure biliary drainage tubes to relieve obstruction and prolong survival; however, the long-term placement of external PTBD tubes can seriously undermine quality of life. Drainage tubes must be replaced frequently due to dislodgement, biliary tract infection, obstruction, and/or cholangitis (12). PTBD tube replacement also imposes the risk of hemorrhage and biliary fistula. This pilot study was the first to combine IRE with intraoperative biliary stent placement for unresectable PHCC. Our primary objective was to destroy cancer cells while maintaining the integrity of the bile duct and thereby eliminate the need for PTBD tubes.

Martin et al. reported preliminary results of using IRE to treat unresectable PHCC (9). In that study, PTBD tubes were indwelled without ERBD stenting for a median period of 122 days. The median PTBD-free period was 305 days; however, nearly all of the patients in that cohort eventually required PTBD tubes to decompress biliary obstruction. This is a clear indication that IRE alone is insufficient to sustain long-term biliary unblocking. Determining whether this was due to tumor recurrence or biliary stenosis resulting from IRE will require further analysis. In the current study, laparotomic placement of biliary stents was performed following IRE (i.e., intraoperatively). In all but one of the patients, serum bilirubin levels presented a significant reduction on POD7, at which time the PTBD tubes were removed. Serum bilirubin levels remained at acceptable levels on POD30 and POD90, and additional external biliary decompression was not required for at least one year after IRE. The one exception involved a complex post-IRE biliary fistula. These results indicate that IRE in conjunction with the intraoperative placement of biliary stents may relieve biliary obstruction in PHCC for a longer time than is possible *via* IRE alone.

IRE alters the permeability of the cell membrane and induces tumor cell apoptosis without inducing thermal injury in connective tissue adjacent to the target lesion. This method has proven to be safe and effective for the treatment of unresectable pancreatic cancer and liver cancer in cases that were deemed unsuitable for other types of thermal ablation therapy (6–8, 13). At the time of diagnosis, the hepatic artery and portal vein (adjacent to the PHCC) are typically invaded by tumors. Under these conditions, thermal ablation therapy is complicated by the risk of vascular injury or incomplete ablation due to the heat sink effect. In the current study, IRE was used for unresectable Bismuth type III/IV PHCC.

Serum CA19-9 levels were significantly higher than preoperative levels on POD3 and significantly lower on POD30 and POD90. Elevated serum CA19-9 levels can probably be attributed to the fact that IRE destroys the cell membrane of tumors, thereby releasing CA19-9 from tumor cells into the bloodstream. Persistently low serum CA19-9 levels on POD30 and POD90 and a complete absence of tumors in follow-up radiological examinations on POD30 provide evidence of complete tumor ablation. This indicates that IRE is well-suited to local tumor control in cases of PHCC. Nonetheless, intraoperative lymph node dissection revealed local lymph node metastasis in 16 patients in the current cohort, indicating that microscopic distant metastasis had already begun at the time of IRE. Note that metastasis occurring outside the ablation zone cannot be controlled by IRE. Most patients in the current study died of distant metastasis; i.e., only three patients developed local recurrence.

For patients with PHCC, complete surgical resection provides the best chance for long-term survival; however, most cases are not amenable to surgical resection. R1 resections are performed in specific cases of locally advanced PHCC. In the current cohort, the median PFS (21.5 months) and median OS (27.9 months) were close to those of R1 resection for PHCC (PFS of 12 months and OS of 24 months) (14). The median OS in the current study (27.9 months) far exceeded the 11.2 months reported for cases of unresectable PHCC in one previous study (15).

In cases of unresectable PHCC, avoiding biliary obstruction and slowing tumor growth are key to prolonging survival. A number of local treatment methods have been combined with biliary decompression for tumor palliation. Gonzalez-Carmona et al. compared photodynamic therapy only (median OS of 15 months) with photodynamic therapy in conjunction with systemic chemotherapy (median OS of 20 months) (16). Moole et al. presented a meta-analysis of photodynamic therapy with biliary stent (median OS of 413.04 days) (17). Endoscopic radiofrequency ablation (RFA) was implemented in conjunction with biliary stents to treat unresectable PHCC, resulting in a mean survival time of 13.2 months and a mean stent patency period of 6.8 months (18). Tan et al. reported on the use of percutaneous biliary stenting in conjunction with radiotherapy for the treatment of palliation unresectable PHCC, resulting in a median survival time of 367 days (19). Taken together, these studies revealed that local treatment in conjunction with internal biliary stents can prolong survival beyond what can be achieved using biliary stents. Nevertheless, palliative local treatments without adjuvant therapy provide a median OS of only 12-15 months, which is far than the median OS of 27.9 months in the current study (16–19).

One limitation of the current study was the lack of uniform adjuvant therapy. IRE provides an acceptable level of local control over unresectable PHCC; however, in most patients, microscopic distant metastasis has already begun at the time of diagnosis, thereby necessitating systemic therapy in addition to local treatment. In the future, IRE could perhaps be implemented in conjunction with adjuvant chemotherapy or immunotherapy to enhance disease control. Another limitation was the small sample size and relative short follow-up period in this study. Furthermore, all of the patients in the cohort were treated at only two medical centers, such that patient selection bias may have confounded the results, and all of the procedures were performed by a small number of surgeons. A multicenter randomized control trial should be conducted to validate the results of this pilot study.

## Conclusions

IRE in conjunction with intraoperative biliary stent placement is a safe and reliable approach to treating unresectable Bismuth type III/IV PHCC with acceptable survival rates. This treatment was shown to decompress biliary obstruction soon after the operation, thereby avoiding the inconvenience and complications associated with external PTBD tubes.

## Data Availability Statement

The raw data supporting the conclusions of this article will be made available by the authors, without undue reservation.

## Ethics Statement

The studies involving human participants were reviewed and approved by Institutional Review Board, National Taiwan University. The patients/participants provided their written informed consent to participate in this study.

## Author Contributions

P-CY and K-WH performed most of the study and drafted the manuscript. P-CY, C-YH, and S-YC participated in the acquisition and statistical analysis of the data. Y-JC, X-YL, QZ, and B-ZZ contributed to the conception and design of this study. B-BC, YG, S-YC, QZ, and S-QH assisted with the interpretation of the results. K-WH and P-CY revised the manuscript for intellectual content. All authors contributed to the article and approved the submitted version.

## Conflict of Interest

The authors declare that the research was conducted in the absence of any commercial or financial relationships that could be construed as a potential conflict of interest.
